# The True Cause of Long and Short-Sightedness; with Observations on the Power to Dilate and Contract the Pupil of the Eye

**Published:** 1859-07

**Authors:** Charles W. Wright

**Affiliations:** Professor of Chemistry in the Kentucky School of Medicine


ART. IV.—*The True Cause of Long and Short-Sightedness, together with a few Observations on the Power to Dilate and Contract the Pupil of the Eye at Will.* By CHARLES W. WRIGHT, M.D., Professor of Chemistry in the Kentucky School of Medicine.

THE cause of long and short-sightedness is attributed to a change in the refracting media of the eye, or to a change in the diameter of that organ, or to both causes combined. Thus, in myopia the refracting power of the humors is said to be too great, by which the image of an object falls short of the retina; whereas in the opposite condition, presbyopia, the refraction is diminished, and the rays of light are not sufficiently converged to depict an image on the retina. In the former condition there is said to be an over-convexity of the cornea and lens, and in the latter a diminished density and quantity in the humors of the eye. In regard to the first of these conditions, there is no proof that the refracting power of the lens, or other humors of the eye, is increased, and in the great majority of cases the cornea is not more convex in near-sighted persons than in those not so affected; on the contrary, the reverse frequently appears to be the case. The only reason for such a supposition is the fact that the sight of the myopic is improved by the use of concave glasses, by which the rays of light are dispersed and prevented from coming to a focus before reaching the retina, or rather the black pigment, which is in reality the surface upon which the image of an object is painted.

Now the essential cause of short-sightedness is to be sought for in the iris, which in all cases is preternaturally contracted, or possesses an unusual degree of irritability. When the case is congenital the iris possesses an excessive degree of sensibility to impressions of light; in fact, this form of the affection is almost always observed in individuals of a nervous temperament, together with an excessive degree of muscular irritability. Exposure to a bright light aggravates the congenital form of the affection by producing contraction of the circular fibres of the iris, and the same remark will apply to large doses of opium, which not only aggravate the affection in the myopic individual, but will produce temporary near-sightedness in a person not so afflicted. In congenital cases any means that will cause dilatation of the pupil, such as belladonna for example, will benefit the patient, and enable him to read with the book held at the same distance as a person would require it whose eyes are sound. I have repeatedly experimented with opium and belladonna, and always with the same opposite results—shortening the field of vision with opium, extending it with belladonna. Snow-blindness is due in great part to powerful contraction of the pupil; short-sightedness it is, in fact, to an excessive degree. It is well known that this affection is much more common in cold climates, where the ground is covered with snow for several months in the year, than it is in warm, cases being frequently met with in the New England States, but rarely in the Southwest.

The true cause of long-sightedness consists in a preternatural dilatation of the pupil of the eye, or, what amounts to the same thing, a loss of irritability of the circular fibres of the iris. This, as is well known, is a condition common to persons advanced in life, or those who are in the habit of viewing objects in the distance. Contrary to the statements of most writers on the subject, the sight of the myopic improves as he advances in life, nor have I ever known such a person to become long-sighted. In senile presbyopia the iris does not respond to the rays of light as readily as it does before that condition obtains. That this condition is to be referred to a loss of power in the iris to respond to luminous impressions, and not to any change in the refracting power of the humors of the eye, or to any alteration in the diameter of that organ, is, I think, sufficiently established by the following experiments.

Thus a large dose of opium, by producing contraction of the pupil, enables the presbyopic to read when the book is held at the usual distance from the eye. Exposing the page to the direct solar rays enables the far-sighted individual to read by powerfully stimulating the iris, when under other circumstances he cannot distinguish a letter without the assistance of glasses. A small piece of card, perforated with a common pin and interposed between the eye and the object viewed, by subserving the purpose of the contracted pupil will enable the individual to distinguish objects at the usual focal distance; the smaller the aperture the shorter the focal distance. In viewing an object through perforated cards, the apertures of which vary in diameter, the pupil remains stationary when the intensity of the light is uniform, as one aperture is substituted for another and the focal distance thereby changed. The perforated card should be held as near the eye as possible in conducting these experiments. Anything that causes an increased flow of blood to the eye, by which the irritability of the muscular fibres of the iris is augmented, will restore the sight. Thus, violent coughing will frequently enable the far-sighted to see objects distinctly at the usual distance for a period of from ten to fifteen minutes.

From the foregoing observations it would appear that the iris is the principal means by which the eye is enabled to adapt itself to viewing objects distinctly which are situated at different distances from the observer. That such is the fact I think there can hardly be a doubt. Thus in a uniform light the sound eye can be made to see objects distinctly at different distances by viewing them through apertures of different diameters, the aperture being diminished as the object approaches the eye. This may be carried to such a degree, by using apertures sufficiently small, that a person will be enabled to read printed matter which is held within half an inch of the cornea. A similar experiment can be made with the iris itself, by allowing the sun's rays to fall directly upon the printed page, when it may be placed in contact with the eyelashes, and the words will be distinctly legible. In such an experiment the pupil is contracted to a diameter less than that of the head of a common pin. Opium will also produce myopia in persons whose eyes are endowed with the usual focal powers, and belladonna, pink-root, and hyoscyamus, produce the opposite effect. The pupil is always observed to expand when a distant object is viewed, and to contract as one near the eye is observed.

As to the power which the ciliary muscle is supposed to possess in adjusting the focal distance of the eye, nothing definite is known; at most, its action is subsidiary, inasmuch as such an arrangement is not at all necessary to the successful operation of the optical mechanism of the eye.

From the foregoing statements it will be observed that the terms *myosis* and *mydriasis* could be very well dispensed with, inasmuch as the former signifies preternatural contraction of the pupil, and the latter dilatation. In other words, myosis is another term for short-sight, and mydriasis for long-sight; the only difference being that these conditions are generally accompanied with some diseased state of the eye. They might with propriety be termed the acute forms of long and short-sightedness. Ergot benefits the sight in a person affected with mydriasis, by producing contraction of the circular fibres of the iris.

As regards the power which man has over the muscular fibres of the iris, there can be no doubt but that by a little practice the pupil can be made to dilate and contract with as much facility, by an effort of the will, as that the number of respiratory movements can be increased or diminished at pleasure. This power over the iris can be acquired by rapidly changing the focal distance of the eye, which can be accomplished by interposing a small object between the eye and a larger one situated a mile or more from the observer. A common pin, held at a distance of four or five inches from the eye and on a line with the distant object, is well adapted to the experiment. On viewing alternately the far and near object, the iris will be observed to dilate when the eye is fixed on the former, and to contract on the latter. On making this experiment a few times the observer will be enabled to contract or dilate the pupil at will; at first, by fixing the eye upon imaginary objects, and in a short time, without any assistance of the kind. The experiment is best conducted in a room where the sun's rays have not a direct entrance, but in other respects the light may be as intense as that which is most agreeable in reading. In a strong light voluntary control over the iris greatly diminishes.

The foregoing remarks embrace the results of a long series of experiments and observations on the subjects discussed, which the author hopes to be enabled to present more fully and clearly at no distant period.

